# Rationale use of Thalidomide in erythema nodosum leprosum - A non-systematic critical analysis of published case reports

**DOI:** 10.1590/0037-8682-0454-2019

**Published:** 2020-09-11

**Authors:** Pugazhenthan Thangaraju, Sajitha Venkatesan, Meenalotchini Gurunthalingam, Shoban Babu, Tamilselvan T

**Affiliations:** 1 Department of Pharmacology, All India Institute of Medical Sciences, Raipur, Chhattisgarh, India.; 2 Department of Microbiology, All India Institute of Medical Sciences, Raipur, Chhattisgarh, India.; 3 Department of Pharmacology, All India Institute of Medical Sciences, Raipur, Chhattisgarh, India.; 4 Department of Pharmacology, All India Institute of Medical Sciences, Jodhpur, Rajasthan, India.; 5 School of information technology, SRM university, Sikkim, India.

**Keywords:** ENL, Management, Severity, Thalidomide, Rationale, Usage

## Abstract

**INTRODUCTION:**

Thalidomide is an anti- tumor necrosis factor alpha (TNF-a) drug used mainly in the management of moderate to severe form of Erythema Nodosum Leprosum (ENL). Because of its teratogenic potential it has to be used under proper supervision. Our critical analysis tries to look into the rationale with which it has been used by means of case reports on lepra reaction.

**METHODS:**

We looked for the case reports between December 2005 to June 2019 in databases like Pubmed, Embase and other relevant resources. We used search words like “erythema nodosum leprosum(ENL)”, “thalidomide”, “case report” in different combinations to get relevant reports that focus on thalidomide usage atleast once at any time point during management. The information extracted were indication of thalidomide use, dose, response, outcome, complication if any, along with all the demographic details and geographical distribution.

**RESULTS:**

We found 41 case reports eligible for analysis.The information was critically evaluated. From the analysis it was found that 7 of the case report mentioned the exact indication, 4 case report showed irrational use of thalidomide in the case of neuritis without use of steroids, 7 showed proper use of Clofazimine prior to thalidomide initiation, 26 case report showed case report of rationale dose range and in 4 case reports clofazimine was used prior to thalidomide along with the rational dose of thalidomide.

**CONCLUSIONS:**

This analysis helps to guide the rationale use of thalidomide focussing on few important points that anyone should keep in mind while managing a case of ENL.

## INTRODUCTION

Erythema Nodosum leprosum (ENL) or type 2 lepra reaction is a serious and difficult to manage immunological activity seen in intermediate borderline lepromatous (BL) and the polar lepromatous leprosy (LL). ENL may present as multiple acute episodes or chronic ENL^1^ ^,^ ^2^. In a large cohort study in India, less than 10% of patients had only a single episode of ENL while around 62.5% had chronic occurring ENL^2^. Various manifestations of ENL include generalized, cutaneous and peripheral nerve involvement. The cutaneous manifestations are widespread crops of erythematous inflamed nodules and papules, which are superficial or deep, that wean off periodically^3^. Ulcerated, necrotic, pustular and bullous forms have also been described. Some nodules lead to fibrosis and scarring^3^. Neuritis present with painful and enlarged nerves with functional impairment. Generalized systemic illness includes high fever and prostration due to immune complex mediated complement activation. Erythema nodosum leprosum is a type III Arthus antigen antibody reaction that causes generalised symptoms throughout the body system ranging from mild to severe spectrum. Edema and transient proteinuria may also occur. Eye involvement in the form of iritis and episcleritis can occur in some cases and may lead to loss of vision. Other features such as pain, photophobia and lacrimation may be absent in contrast to the other bacterial infections of eye^3^. Orchitis, lymphadenopathy, organomegaly, joint involvement, dactylitis and bone tenderness over the tibia, are well recognized and documented features of ENL. The chronic type of ENL is difficult to manage in the peripheral set up and always needs referral to tertiary or higher centers. There is a general opinion among the treating physicians that 1 log reduction of bacilli will take 1 year for bacilli to be cleared and in most of the cases the ENL severity correlates with this bacillary index^4^. This chronicity also has an impact on the psychological status of the patients with lepra reaction that will aggravate the existing conditions^5^.

### Currently available treatment for ENL

The main objectives in the management of ENL are to control the inflammation, pain relief and prevention of further episodes^6^. Mild cases are treated with Non Steroidal Antiinflammatory Drugs(NSAIDs) like aspirin and paracetamol. In moderate and severe cases, corticosteroids are the mainstay of treatment. The most commonly used corticosteroid is oral prednisolone. Oral Prednisolone is started at the dose of 40-60 mg /kg body weight/ day to a maximum of 1 mg/kg/body weight/ day. The dose is tapered gradually with the improvement of symptoms up to 5 mg and stopped once the reaction subsides^7^.Majority of the patients require multiple courses of prednisolone due to the natural history of the disease^2^. In steroid dependency or steroid induced complications, Clofazimine is used. Clofazimine is a dye acting as an anti-inflammatory and antimicrobial agent. But it has a slower onset of action^8^. Clofazimine at a dose of up to 300mg daily can be given to control ENL. This higher dose should not be continued for more than 12 months. Treatment with thalidomide has been useful to reduce the prednisolone requirement of patients with chronic ENL^9^. Due to its teratogenicity potential, Thalidomide should be used with extreme caution in reproductive age group of both the sexes even though WHO doesnot recommend it^10^. The Risk Evaluation and Mitigation Strategy (REMS) program adopted by the USFDA has been useful in preventing pregnancies in women taking thalidomide^11^. Because of its good efficacy in managing the severe cases of ENL, it is still being used as a drug in emergency conditions where steroids or other drugs are contraindicated. In this background, ensuring proper and rational use of thalidomide becomes the responsibility of the treating physician. 

Rational use of medicines in this context may be defined as the patients receiving medications relevant to their clinical needs, in doses that meet their own individual requirements, for an adequate period of time, and at the lowest cost to them and their community. It is also mandatory to know the 12 key interventions that the WHO advice for promotion of rational use of drugs^12^ like establishing national level multidisciplinary body for coordinating the policies on medicine use, using available clinical guidelines effectively, developing and using the medicines enlisted in the National List of Essential Medicines, forming committees for drugs and therapeutics in the district hospitals, including the problem-based pharmacotherapy in the undergraduate curriculum, to continue in-service medical education as a requirement for obtaining license for practising, routinely supervising and auditing and obtaining feedback from the practitioners, using information of medicines independently, creating awareness and educating the public about medicines, avoiding financial incentives perversely, enforcing appropriate regulation and ensuring availability of staff and medicines by sufficient funding by the Government.

So to know the rationale behind the treatment, there is an increasing need to describe the various treatment procedures that will develop the understanding of readers of published content and treatment protocol. It is also mandatory to describe the type of drug used, route of administration, approved dosage and frequency, possible side effects. Case reports were studied in this review as detailed information regarding treatment course and characteristics are mentioned irrespective of severity and presentation of ENL. It also helped us explore if drug use was rational. Moreover, case reports are not planned and structured study as in the case of randomised clinical trials. They are done based upon the knowledge and experience of the treating physician which helps us get an insight about how far the treating physicians are aware of the treatment protocol and guidelines while using Thalidomide for ENL.

## METHODS

### Search strategy

We identified case reports of the following bibliographic databases MEDLINE, Embase and Web of Science. In PubMed the case reports were identified by using searches included as (lepra[All Fields] AND reaction[All Fields] AND ("thalidomide" [MeSH Terms] OR "thalidomide"[All Fields])) AND (Case Reports[ptyp] AND ("2005/01/01" [PDAT] : "2019/06/30"[PDAT])) (("erythema nodosum"[MeSH Terms] OR ("erythema"[All Fields] AND "nodosum"[All Fields]) OR "erythema nodosum"[All Fields]) AND leprosum[All Fields] AND ("thalidomide" [MeSH Terms] OR "thalidomide"[All Fields])) AND (Case Reports[ptyp] AND ("2005/01/01"[PDAT] : "2018/06/30"[PDAT])). In addition, we also searched references of the studies of screened articles to extract information about relevant articles.

### Study selection

In the first step, titles and abstracts of the articles were reviewed and irrelevant articles were excluded. In the next step, full texts of the selected articles were screened as per inclusion and exclusion criteria.

### Inclusion criteria

(a) Patients: any age group and both genders 

(b) Intervention: thalidomide used at anytime point

(c) Type of study: case reports/case series. 

### Exclusion criteria

Articles of ENL where thalidomide is not used were excluded from the study.

### Data extraction (selection and coding)

Two reviewers have independently screened the titles and abstracts of the references retrieved in the searches to identify potential reports that meet the inclusion criteria, before screening the full-text papers. The information on authors, patient demographic like age, sex, area of residence, clinical aspects of disease, investigation and special investigation, diagnosis and the treatment of the conditions with outcome, additional remarks if any and their year of publication and journal were extracted. We focused only on the treatment part which contains Thalidomide and the points relevant to it.

Regarding thalidomide the information’s extracted include rational indication, dose at start of reaction and average daily dose, and outcome of the treatment. The outcome is called “responded” if there is clinical response to the use of thalidomide in reversing the severity of symptoms of reaction and complete remission from the start of initiation of thalidomide and sustained for several months to year. Rationale use of thalidomide is based on the guidelines of various countries (Brazil^13^, Japan^14^, USA^14^) and reaction management program adopted. Japan adopted the USA guideline with little modification with respect to the Dose. Regarding Brazil, even though strict special recommendation has been in management protocol of ENL, still cases of embryopathy and baby born with phacomelia is seen^13^. The countries in Europe also follow similar recommendation of dosage of 300 mg and indications as given by the recommendation by USA and its manufacturer^14^.

 The factors essential for rationality are the proper indication of starting the drug and the approved dose as per the symptoms. There are exceptions where it is directly started based on case to case variation. The approved indication and the dose for treatment of severe ENL is 100 - 400 mg/day in divided doses shown in (Table 1)^14^. 

**TABLE 1:** Approved indication and dosage^14^.

INDICATIONS
Acute treatment- cutaneous manifestations- moderate to severe erythema nodosum leprosum (ENL).
Not indicated as monotherapy - Presence of moderate to severe neuritis.
Maintenance therapy- cutaneous manifestations of ENL recurrence
DOSAGE AND ADMINISTRATION
100 to 300 mg/day OD or in divided dose.
Initiate dosing at the low end of the dose range for less weighing patient.
Higher dosage range - severe cutaneous ENL reaction, or in those who have previously required higher doses to control the reaction (up to 400 mg/day)
Concomitant use of corticosteroids - moderate to severe neuritis.
Continue dosing at least 2 weeks.
Patients may then be reduced of medication in 50 mg decrements every 2 - 4 weeks.
Reduction should be attempted every 3 - 6 months and in reduction of 50 mg every 2 to 4 weeks.

### Statistical analysis

All the information from the case reports were extracted in the excel sheet. We focused on the information pertaining to thalidomide use, like indication, dose and its combination and adverse events. Data was presented in numbers and percentages.

## RESULTS

**FIGURE 1:**
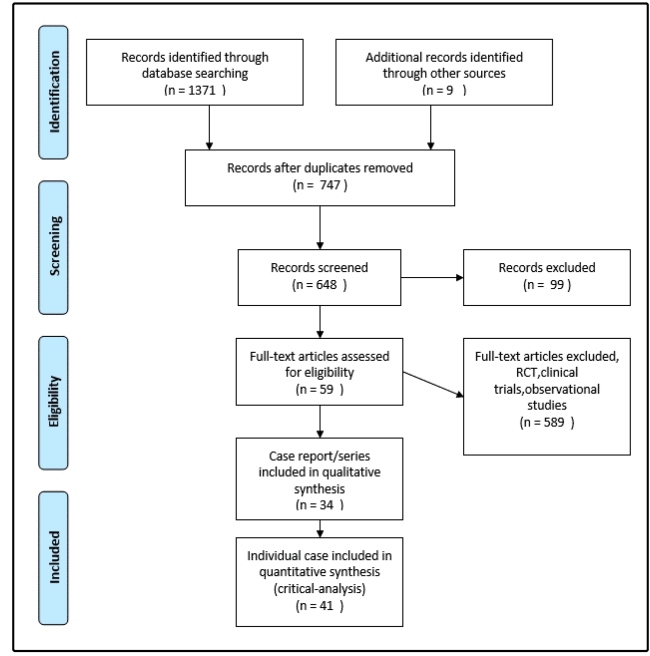
Flow diagram of case reports included in the review(PRISMA).

A total of case report/series reported from 01-01-2005 to 30-06-2019 were 35 from PubMed and 9 from other series. A total of 34 case report/ series which met the inclusion criteria were screened and selected for analysis. There was a total of 41 individual cases (Figure 1) reported in the 34 available case report/ series. Leon et al had 2 cases^15^, Fogagnolo et al had 2 cases^16^ , Rahul nagar et al had 3 cases^17^ and Rattan et al had 4 cases^18^ ^-^ ^20^, in their case series summing up to 41 case reports in 34 case series. 

Out of the 41 cases reported (Table 2), 30 were male and 11 were female with 26 reports are from India and the remaining 15 were from outside India.The distribution of 7 reports from Brazil, 1 from Phillipines, South East Asia, USA, Columbia, Netherlands, China and Japan each (Table 2 **/** Supplementary Figure 1). 

Regarding the age distribution (Table 2), there were no cases reported in the age group of 1-10 years. The highest number of 13 cases were reported in the age group of 31-40 years, out of which 9 were males and 4 were females. The next highest number of 11 cases were detected in the age group of 21 -30 years where 10 were male and 1 was female. 5 cases were detected in the age group of 41-50 years where 4 were male and 1 was female. 6 cases were detected in the age group of 51-60 years where there was equal distribution among both sexes accounting for 3 each. 3 cases (all male) were detected in 61- 70 years age group and 2 cases ( all female ) were detected in the age group of 11-20 years. 

With respect to the Bacterial Index (BI)(Table 2), it was not mentioned in 18 cases. Out of the remaining 23 cases, BI was mentioned as 1+, 2+, 3+, 4+,5+ and 6+ in 1,1,3,4,4,10 cases respectively. 

**TABLE 2:** Demographic details.

Demographic parameters	Number
Age	
1-10	0
11-20	2
21-30	11
31-40	13
41-50	5
51-60	6
61-70	3
Sex	
Male	30
Female	11
Geographical distribution	
India	26
Brazil	7
Philippines	1
South east Asia	1
USA	1
Micronesia	1
Columbia	1
Netherlands	1
China	1
Japan	1
Bacillary index	
NA	18
1+	1
2+	2
3+	3
4+	3
5+	4
6+	10

**NA:** Not Available.

With respect to rational use of Clofazimine and thalidomide (Supplementary Table 1) only 7 cases has been found out to be a rationale. 6 out of the 7 cases were from India and 1 from outside India. 

With respect to the rational use of thalidomide (Table 3) in proposed dosage 26 cases has been found out to be a rationale. Out of the 26 cases 18 were reported from India and the remaining 8 from outside India. There were two cases of ENL in which thalidomide is planned as per the existing protocol and they were from India (Table 3).

**TABLE 3:** Rational use of Thalidomide along with Clofazimine with reference to individual countries.

S.no	Reference	Country	Sample	Treatment	Clofazamine	Rationale-(Thalidomide
			size		Y=1 N=0	clofazamine) Interpretation
1	Fogagnolo et al^16^	Brazil	2	Thalidomide 300mg/day, Prednisolone 80mg/day	0	No-Not always mandatory
2	Rahul nagar^17^	India	3	I- Prednisolone 40 mg/day	1	Yes
				clofazimine 300 mgs/day		
				thalidomide was tapered below 100 mgs/day.		
						
				II- Prednisolone 40 mgs/day,clofazimine 100 mgs three times a day prednisolone in higher doses of 60 mgs/day. Two successive thalidomide courses were also found	1	
				III- Prednisolone 40 mgs/day,clofazimine 100 mgs three times a day prednisolone in higher doses of 60 mgs/day. Two successive thalidomide courses were also found	1	
3	Rattan et al^18^	India	4	Thalidomide 100mg/thrice a day + MDT - MB	0	No
				Thalidomide 100mg/thrice a day + MDT - MB Thalidomide 100mg/thrice a day + MDT - MB		
				Thalidomide 100mg/thrice a day + MDT - MB		
4	Mahajan et al^21^	India	1	Treatment restarted with prednisone (60 mg/day), clofazimine (100 mg three times/day), rifampicin (600 mg once a month), thalidomide (100 mg three times/day)	1	Yes
5	Jitendra ssv et al^22^	India	1	Prednisolone 1 mg/kg/day	1	Yes
				Clofazimine 300 mg/day for six months and 100 mg/day for another six months		
				Thalidomide 200 mg/day (STOPPED)		
6	T . Narang et al^23^	India	1	Started rationale dose but patient not tolerated (apremilast)	1	Yes
7	Barman et al^24^	India	1	MDT, Thalidomide - 100mg, T. Prednisolone.	0	No
8	Richards^25^	Pt from Micronesia- treated at Dallas, Texas	1	MDT, Prednisolone 60mg/day, Thalidomide - 100mg	0	No, Not always mandatory
9	Rodriguez^26^	Colombia	1	MDT, Thalidomide 300mg/day, Prednisolone 50mg/day	0	No, Not always mandatory
10	Verma et al^27^	India	1	MDT, Prednisolone 40mg/day, Thalidomide 100mg TDS, Azathiprine 50mg/twice daily.	0	No
11	Verma et al^27^	India	1	MDT,Prednisolone 1mg/kg/day, Thallidomide 100mg QID for 4 months	0	No
12	Yogeesh HR et al^28^	India	1	MDT, Prednisolone 30mg, Thalidomide 100mg TDS,	0	No
13	Faber et al^29^	Netherlands	1	MDT, Steroids, Thalidomide 300mg, Pentoxiphylline 400mg, Finally, Infliximab 5mg/kg.	0	No, Not always mandatory
14	Hebe Petiti-Martin et al^30^	Spain	1	MDT, Prednisolone, Thalidomide - 150mg/day [stopped], Enoxeparin, Acenocoumoral	0	No, Not always mandatory
15	Vetrichevel et al^31^	India	1	MDT, Corticosteroids, Thalidomide 300mg/day then stopped, LMWheparin, Warfarin for 6 months.	0	No
16	Sundeep chowdary et al^32^	India	1	Prednisolone (40-80 mg)	1	No
				Etanercept (50 mg / week) subcutaneously.		
				Clofazimine (300 mg daily),		
				Thalidomide (starting dose: 300 mg daily),		
				Minocycline (100 mg daily),		
				Clarithromycin (1 g daily),		
				Ofloxacin (400 mg daily),		
				Pentoxifylline (400 mg three times daily),		
				Azathioprine (150 mg daily; later increased to 300 mg)		
17	Julia Rocha Silva Santos et al^33^	Brazi	1	Thalidomide (100- 300 mg/day)	0	No, Not always mandatory
		l		prednisone at (20 - 40mg/day)		
				50mg of etanercept SC.		
				Thalidomide was interrupted		
				Prednisone was continued at a dose of 10mg/day		
18	Gulanikar et al^34^	India	1	Prednisolone 30mg . Thalidomide 100mg QID	0	No
19	Yamaghuchi et al^35^	Japan	1	PSL 15-55 mg⁄ day,Thalidomide 100-300 mg⁄ day	0	No, Not always mandatory
20	Sharma et al^36^	India	1	MDT	0	No
				Prednisolone 60 mg/d,		
				Ibuprofen 400 mg t.i.d.		
				colchicine 0.5 mg b.i.d.		
				thalidomide 100mg bd and **stopped**		
Rationale with respect to use of thalidomide in planned dose:						
21	Satyendra Kumar Singh et al^37^			Plan for Thalidomide in desired rationale dose		
22	Prasan Kumar Panda et al^38^			Plan for Thalidomide in desired rationale dose		

The major indication for starting thalidomide (Table 4) is severe ENL or recurrent ENL. This was mentioned only in 7 cases and the in the rest of the 35 cases, there was no mention of the severity of ENL but merely ENL.

With relevance to neuritis and thalidomide (Table 4), out of the 41 cases, there was no neuritis in 21 cases. In 16 cases, thalidomide had been prescribed along with steroids in a rationale indication. In 4 cases, steroids were not given along with thalidomide.

The duration of treatment with thalidomide is divided into treatment less than 3 months, for 3 months and more than 3 months. Generally the range of duration falls between 3 months to 6 months based on the dosage the drugs was being started and the tapering once in two weeks. As general 3 months is ideal duration a patient can respond after due tapering and stoppage. But it depends on case to case basis (Table 4).

Regarding the dose (Table 4), Out of the 41 cases studied, the dose of thalidomide was not mentioned in detail in 15 cases. The dose of 100 mg, 150 mg, 200 mg, 300 mg and 400 mg was given in 3 cases, 1 patient, 2 cases,18 cases and in 2 cases respectively. 

Out of the 41 cases 24 cases responded to the treatment with thalidomide whereas in 17 cases thalidomide was stopped (Table 4).

**TABLE 4:** Details of Thalidomide usage.

Thalidomide	Number
**Severity mentioned**	
ENL	35(83)
Severe ENL	7(17)
**Duration of treatment**	
Not mentioned	15(35.71)
< 3 Months (treatment stopped)	6(14.29)
-3 months	17(40.48)
> 3 months	3(7.14)
**Thalidomide and steroids indications**	
No neuritis	21(51)
Neuritis- Thalidomide + steroids	16(39)
Neuritis- Thalidomide alone	4(9.75)
**Thalidomide dose**	
Not mentioned	15(36.58)
100MG	3(7.32)
150MG	1(2.44)
200MG	2(4.88)
300MG	18(43.90)
400MG	2(4.88)
**Outcome**	
Responded	24(59)
Stopped	17(41)
**Reasons for stopping Thalidomide**	
NR-other drugs used	8(47)
Peripheral neuropathy	2(12)
DVT	4(23)
Pregnancy	1(6)
Intolerance	1(6)
Died	1(6)

The reasons for stopping Thalidomide may be due to drug intolerance, pregnancy, non responsiveness to the drug or adverse drug reactions.

Out of the total 41 cases, thalidomide was stopped in 17 patients after starting the treatment (Table 4). In 8 patients there was no response to Thalidomide and there was subsequent use of other drugs like Methotrexate, Etanercept, Azathioprine and Infliximab were used. Thalidomide was stopped due to peripheral neuropathy in 2 patients and due to deep vein thrombosis (DVT) in 4 patients. Among the 4 patients who developed DVT due to thalidomide, one patient was given Etanercept. One patient became pregnant after the start of the treatment with thalidomide and therefore the drug was withdrawn owing to its teratogenic effect. One patient died during the course of treatment not due to thalidomide but due to associated septicaemia.

Out of all the case reports, only 4 case reports met the rational criteria for usage of Thalidomide in the recommended dose and was used after using Clofazimine. All the 4 case reports were from India.(Table 3 **/** Supplementary Figure 1).

## DISCUSSION

This is the first analysis of its kind which focuses on the rational use of thalidomide in ENL in the published literature till 30-06-2019. The analysis was made using the case reports/series. The objective of selecting case report is that the rationale use can be only assessed in the case report from various part of the world. As already commented, case report are not a planned work with which a physician manages. The treating physicians manage the case as per their knowledge in the existing scenario and protocol and guideline but not necessarily to compile data as in the case of planned structured studies. Hence, we felt critical analysis would help in bringing out information at the treating level of health professionals. 

According to the WHO’s definition of rational drug, the drug should be enlisted in the National List of Essential Medicines and awareness should be created among the public regarding its use. As per the analysis, it was found that thalidomide is enlisted in the national list of essential medicines^39^ but not for the ENL indication. But awareness has been created to those specifically involved in the management of lepra reactions through country programme and the institutes managing the severe cases of ENL.

The WHO does not support the approved use of thalidomide in the management of severe ENL as thalidomide being a proved teratogenic^10^. Although the legislations and laws cover for restricted sale and supply of thalidomide, dispensation of thalidomide being active in Brazil has caused recent registering of cases of thalidomide embryopathy^40^ ^,^ ^41^.

But it is off the document that the countries harbouring leprosy burden, thalidomide is being used with all necessary precautions for the treatment of ENL as per the indication. Various countries like the United States of America, the European countries , India, Brazil^42^ and Japan^43^ use Thalidomide for the treatment of ENL following the US- FDA^44^ recommendation ie. Thalidomide in a dose of 100-300 mg. 400 mg of thalidomide given in severe cases of ENL. 

It is being used for this specific indication only in the speciality centres that was approved for usage with proper registries like Central Leprosy Teaching And Research Institute (Government of India), many NGO under the leprosy mission, Bombay projects etc. 

From the total 41 cases analysed, it was found that there was a male predominance over the females where 73% i.e. 30 were males and 27% i.e. 11 were females. A major chunk of 67% i.e. 26 cases were reported from India followed by Brazil with 7 cases and the rest 8 cases have been reported from various other parts of the world.

When we consider the age distribution of severe ENL reported, it was found that the majority of cases were reported in the age group belonging to 21-40 years again with a male preponderance. There were a total of 24 cases in the age group of 21-40 years out of which 11 were reported in 21-30 years of age group and 13 in 31-40 years of age group. It might be due to the fact that the immune system namely the humoral component is active in the age group of 21-40 years leading to a majority of ENL cases reported in this age group. It may also be due to the fact that it is productive age group where the patients may be losing their productive days due to restricted movements which may lead to decrease in their ability to earn income. It is to be noted from the case studies that higher the Bacterial Index (BI) higher is the chance of recurrent ENL and it is an already established fact and has been published in literatures.

Out of the 41 case studies, only in 7 cases the severity of ENL has been mentioned (severe/ recurrent). In the remaining 34 cases there is no mention of severity. As per the guidelines/protocol, thalidomide is indicated only in severe or recurrent ENL. According to this analysis it is clear that there is need for proper mentioning of the indication of the severity of ENL as it is the deciding criteria for using thalidomide. 

Thalidomide should be used in case of neuritis only along with steroids^44^. In our study we found that out of the total 20 cases where peripheral neuritis was reported, thalidomide was used along with prednisolone in 16 cases. In the remaining 4 cases thalidomide was used alone against the guidelines where it should be used with corticosteroids. 

The approved dose range is between 100 to 400 mg as a divided dose^44^. The dose of thalidomide was not mentioned in 15 cases. This should be always mentioned as it will help the clinicians and the readers to find whether it is because of non availability during the course of treatment, or non responsiveness to low dose range of approved dosage so the case is managed with alternative drugs and other possible reasons.The protocol of reduction of doses depends upon the clinical improvement on a case to case basis. The dose may be reduced by 50 mg decrements every 2 - 4 weeks. This reduction should be attempted every 3 - 6 months^44^.

According to the treatment guidelines management of ENL by WHO^45^, a case of severe of ENL should be first treated with Prednisolone in adequate doses. If the patient did not respond to prednisolone then the patient is put on Clofazimine only or in most cases a combination of Clofazimine and prednisolone regimen. In cases where the patients do not respond to either Clofazimine or Clofazimine and prednisolone combination, then the patients are considered for treatment with thalidomide. The Clofazimine is used as one capsule of 100 mg 3 times a day for initial 3 months followed by one capsule of 100 mg 2 times a day for the next 3 months and finally one capsule of 100mg once a day for next 3 months. Even though Clofazimine is less potent than steroids and often takes 4 - 6 weeks to develop its full effect; it is extremely useful in reducing or withdrawing corticosteroids in patients who have become dependent on them. Total duration of Clofazimine therapy should not exceed 12 months.

Among the 41 case studies, Clofazimine was started or given in only 7 cases. In 26 case studies thalidomide was used rationally with respect to dose. But among all the 26 cases where thalidomide was given rationally with respect to the dose, only 7 case studies showed that the patients were started with Clofazimine before using thalidomide. Regarding the usage of thalidomide after Clofazimine, it is the standard protocol being practiced in India unless contraindicated. Even among Indian studies, only 7 out of 18 cases followed the protocol of Clofazimine and thalidomide. In other countries, because of fairness in skin colour, Clofazimine is seldom used either in MDT regimine or in management of lepra reaction because of hyperpigmentation.

Out of the total 41 cases, thalidomide was stopped in 17 patients after starting the treatment. In 8 patients there was no response to Thalidomide and there was subsequent use of other drugs like Methotrexate, Etanercept, Azathioprine and Infliximab were used. Thalidomide was stopped due to peripheral neuropathy in 2 patients and due to deep vein thrombosis (DVT) in 4 patients. Among the 4 patients who developed DVT due to thalidomide, one patient was given Etanercept. One patient became pregnant after the start of the treatment with thalidomide and therefore the drug was withdrawn owing to its teratogenic effect. One patient died during the course of treatment not due to thalidomide but due to associated septicaemia. There was no mention of the precaution or advice of precaution which has to be given mandatorily to all female patients of reproductive age group.

The analysis of case report has widened our eyes in various aspect of erythema nodosum leprosum management. There is a need by the physcician who were instrumental in treating the reaction case for detailed description of case, categorise the severity and the treatment as per their severity scale. We also found that in spite of rationale usage of thalidomide also there are cases not responded with the approved dose and frequency explaining the strong immunological response or inflammation that needs other immunomodulatory and biologics. This analysis also shows a major gap in following the treatment guideline wherein the treating physicians are lacking the proper information regarding the use of thalidomide and clofazimine in the management of ENL or it is the severity of ENL that makes the physician not to wait with slower acting drug clofazimine and directly dispensed the thalidomide or non availability of clofazimine that warrents to use thalidomide. Hence there is a need to spread proper awareness among the treating physicians for treatment of severe cases of ENL. But in countries like Brazil, thalidomide is considered as the drug of choice for managing severe ENL at the time of initiating management. The same Brazilian health ministry also recommends Clofazimine and prednisolone. So country to country variability should also be taken into consideration.

From the analysis, we found that there is lacuna in the rational usage of thalidomide with respect to indication, dose and usage along with clofazamine and corticosteroids. But clofazimine usage depends upon geographical distribution based on its character of causing hyperpigmentation.So outside India it is seldom used and alternative of pentoxyfylline or minocycline is being tried. There is a need for proper guideline for usage of thalidomide and awareness should be created among the treating physicians for the same. If rationally used, thalidomide is a boon in the treatment of severe ENL.

## RECOMMENDATIONS

- Case report pertaining to ENL should mention the severity scale of ENL
- Listing of other drugs used with proper dose and duration before starting thalidomide should be explained in detail.
- Proper use of steroids and thalidomide in case of neuritis should be stressed.
- Thalidomide with approved dose and frequency should be used.
- Usage of newer biologics in ENL should be sufficiently followed up.
