# Supplementary material for: Rationale use of Thalidomide in erythema nodosum leprosum - A non-systematic critical analysis of published case reports
Source: Rev Soc Bras Med Trop. 2020 Sep 11;53:e20190454. doi: 10.1590/0037-8682-0454-2019 (PMC7491565; doi:10.1590/0037-8682-0454-2019)
Supplement: Supplementary file 1 [file 1678-9849-rsbmt-53-e20190454-suppl1.pdf]

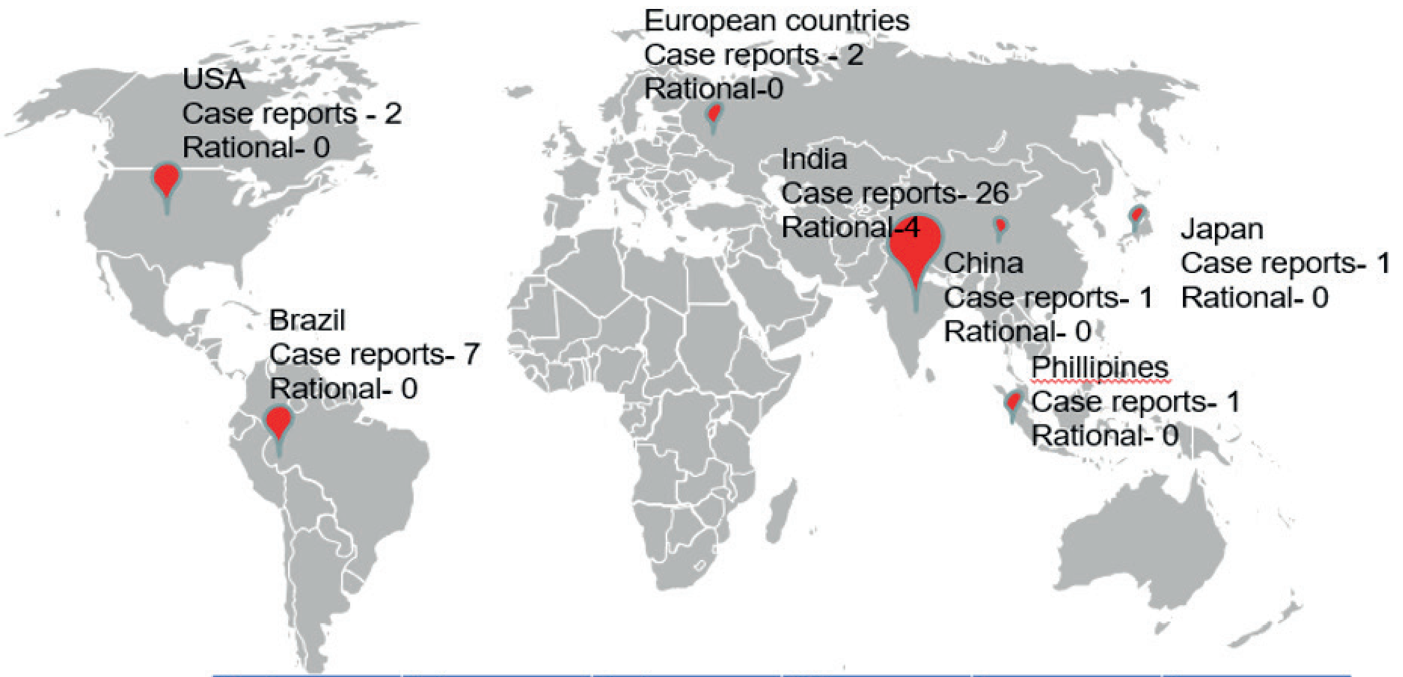

Country	India	Brazil	USA	Japan	Europe
Thalidomide	100-300 mg	100-300 mg	100-300 mg	50-300 mg	100-300
Maximum (in severe ENL)	400 mg	400 mg	400 mg	400 mg	400 mg

SUPPLEMENTARY FIGURE 1: World map showing case report distribution worldwide and the rational use of thalidomide in the case reports.
