# Supplementary material for: Rationale use of Thalidomide in erythema nodosum leprosum - A non-systematic critical analysis of published case reports
Source: Rev Soc Bras Med Trop. 2020 Sep 11;53:e20190454. doi: 10.1590/0037-8682-0454-2019 (PMC7491565; doi:10.1590/0037-8682-0454-2019)
Supplement: Supplementary file 2 [file 1678-9849-rsbmt-53-e20190454-suppl2.pdf]

SUPPLEMENTARY TABLE 1: Rationale with respect to use of clofazimine in proposed dose and duration:

S.NO	AUTHORS	TREATMENT
1	Rahul nagar et al[17]	I - Prednisolone 40 mg/day along with clofazimine 300 mgs/day thalidomide was tapered below 100 mgs/day.
		II - prednisolone 40 mgs/day, clofazimine 100 mgs three times a day prednisolone in higher doses of 60 mgs/day. Two successive thalidomide courses were also found
		III - 60 mgs/day prednisolone and 300 mgs/day clofazimineTwo following courses of thalidomide
2.	Shah et al[19]	MDT + Oflox + Prednisolone. Ofl stopped, Clofazimine increased, Thalidomide included.
3.	Mahajan VK[21]	Treatment restarted with prednisone (60 mg/day), clofazimine (100 mg three times/day), rifampicin (600 mg once a month), thalidomide(100 mg three times/day)
4.	Jitendra ssv et al[22]	oral prednisolone 1 mg/kg/day<br>oral clofazimine 300 mg/day for six months and 100 mg/day for another six months. Thalidomide 200 mg/day (STOPPED)
5.	T . Narang et al[23]	After sufficient dose of clofazimine
